# Fellowships, Grants, & Awards

**Published:** 2005-09

## Tools for Zebrafish Research

This Program Announcement (PA) is to encourage investigator-initiated applications designed to exploit the power of the zebrafish as a vertebrate model for biomedical and behavior research. Applications proposing to develop new tools or genetic or genomic resources of high priority to the zebrafish community that will advance the detection and characterization of genes, pathways, and phenotypes of interest in development and aging, organ formation, behavior, and disease processes are welcome. This effort stems from an NIH initiative developed by the Institutes and Centers of the Trans-NIH Zebrafish Coordinating Committee (TZCC; http://www.nih.gov/science/models/zebrafish/) under the co-chairmanship of NICHD and NIDDK. Since its formation in 1997, the committee has played an active role as an advocate for the zebrafish as an important model for development and disease research. PAR-02-142, “Tools for Genetic Studies in Zebrafish” (http://grants.nih.gov/grants/guide/pa-files/PAR-02-142.html) was issued in August 2002 because it was clear that there was a critical need for non-hypothesis driven, tool development proposals to be reviewed as a group, within a single framework. It focused on identifying additional mutants and developing new genetic tools in zebrafish. Ongoing dialog with the zebrafish research community, most recently at the Sixth International Meeting on Zebrafish Development and Genetics in June 2004, suggested a continuing need for not only tools, but high priority resources as well. Therefore, this PA is a continuation of the program initiated by PAR-02-142. The objective is to continue to broaden the range, power, and utility of tools for biomedical and behavioral research using zebrafish, and to develop genetic and genomic resources of high priority to the zebrafish community. Methodology developed and data and mutants generated as a result of this PA are expected to be made widely available to the research community as described by NIH Grants Policy (Principles and Guidelines for Recipients of NIH Research Grants and contracts on Obtaining and Disseminating Biomedical Research Resources: Final Notice, December 1999, http://ott.od.nih.gov/NewPages/Rtguide_final.html and the NIH Model Organism Sharing Policy, http://grants.nih.gov/grants/policy/model_organism/index.htm).

Objectives to be addressed in applications submitted in response to this PA include, but are not limited to, the following: 1) development and/or application of novel methods of mutagenesis (e.g., insertional, site-specific, conditional knockout vectors or systems); 2) development of techniques supporting more efficient targeting of induced local lesions in genomes (TILLING); 3) development of technologies for gene inactivation and for gene expression manipulation including, but not limited to, morpholino oligonucleotides, new types of antisense technology, techniques for homologous recombination, techniques for gene trapping, and strategies for directing gene misexpression, or other transgenic methodologies; 4) development of high throughput small molecule screens; 5) development of new genetic or genomic resources that are of high priority for the zebrafish community; 6) development and/or application of novel screens for mutants; these may be refinement of phenotypic analyses preparatory to screening, or phenotypic screens based on observation of alterations in morphology, physiology, or behavior; 7) screens focusing on identifying novel developmental genes; and pathways, including those mediating sensitivity or resistance to environmental toxicants; 8) screens to analyze the genetic basis of adult phenotypes including behavior, aging, organ disease, cancer, and responses to environmental toxicants, alcohol, and drugs of abuse.

The participating NIH Institutes and Centers have provided a brief outline of their interests as they relate to the goals of this PA. These brief mission statements are intended to indicate the breadth of the biomedical areas of interest in which zebrafish are likely to be a useful model.

NCI: Generation and study of zebrafish models to identify and place genes in functional pathways that effect growth and development, in particular, genes/pathways that, when altered, result in uncontrolled or cancerous growth.

NCRR: The NCRR supports research projects that broaden the utility of the zebrafish model for cross-cutting biomedical research that is not encompassed within a single NIH Institute or Center. Interests include, but are not limited to, development of new methods for mutagenesis and/or phenotypic characterization that would be of use in research on a wide range of diseases or organs, particularly if these methods could be applied to other animal models as well as the zebrafish.

NEI: Research on the normal and abnormal visual system, including eye development, optic nerve guidance and the visual centers of the brain. This research might include the use of mutants to elucidate the cellular and molecular processes that control normal eye development and function and to provide models for the investigation of the genetic bases of inherited eye diseases.

NHLBI: Cellular and molecular functions of zebrafish genes that have potential to model human cardiovascular, blood, and pulmonary, or sleep disorders. Genetic basis of disorders of cardiovascular development and function; effect of mutations on subsequent organ development leading to such disorders as arrhythmia, cardiac hypertrophy, dilated cardiomyopathy, and heart failure; developmental aspects of endothelial dysfunction as the basis for vascular disorders; developmental defects in hematopoiesis and the relationship to disorders of the hematopoietic system; genetic basis of angiogenesis, and vasculogenesis; and, the genetic basis, regulation, and role of biological clock mechanisms in development and circadian behavior.

NIA: Basic research on the genetic and molecular basis of aging and longevity. Generation and analysis of late-onset disease models or long-lived mutants that can be used to identify, clone, and characterize genes involved in normal and pathological aging. Cellular and molecular function of genes expressed, for example, in the aging nervous system, cardiovascular, immune, and musculoskeletal systems. Such genes include, but are not limited to, those involved in neurodegenerative disorders, neuroplasticity, cell death, damage and repair of DNA and proteins, oxidative stress, and maintenance of differentiated cell function.

NIAAA: Mechanistic studies of ethanol-induced teratogenesis, behavioral impairments, and organ damage. These studies may include screening methods for alcohol-related phenotypes, gene identification, and functional analyses of these genes.

NIAMS: Mutations that have the potential to illuminate the development and function of the vertebrate musculoskeletal system and skin. The musculoskeletal system includes muscle, bone, articulated joints, cartilage, tendon, and ligament. Priority will be given to the establishment of collaborations between investigators with expertise in the zebrafish and investigators with expertise in the musculoskeletal systems and skin of mammals and humans.

NICHD: Identification, cloning, and characterization of the genes important in normal development as well as those mutant genes that cause developmental defects. Elucidation of the cellular, biochemical, molecular, and genetic mechanisms underlying normal and defective development. This includes, but is not limited to, the study of general mechanisms of pattern formation and cell lineage, neural crest development, cell specification, differentiation, migration, and fate in early development of many organs/systems such as limb, nervous system, immune system, and heart.

NIDCD: Identification and cloning of genes/proteins involved in the normal and disordered development in the areas of hearing, balance, smell, taste, voice, speech, and language. Elucidation of the cellular, molecular, and biochemical and sensory processing mechanisms governing the proliferative, regenerative, lineage determination, and developmental capacities of these sensory cells and tissues.

NIDCR: All aspects of normal and abnormal craniofacial development, including genetics, complex origins of craniofacial disorders, cell lineages and differentiation, cell signaling and gene regulation, embryonic patterning, imaging, biomimetics, and new technologies for high-throughput genetic and protein screens.

NIDDK: Research on diabetes, particularly studies on pancreatic beta cell function and development, obesity and mechanisms underlying satiety, other endocrine and metabolic diseases, hematologic disorders, physiology and diseases of the digestive system, liver, kidney, and urinary tract. Studies aiming to clarify the cellular and molecular events that dictate tissue and organ formation in all these systems are considered of relevance. In addition, studies that exploit the zebrafish to model physiological processes such as renal function, fluid and electrolyte balance, are relevant to NIDDK. These studies could include, but need not be limited to, studies to develop cell lines from any of the tissues or organs of interest, studies to characterize normal or abnormal function of tissues or organs of interest, methods to screen and identify additional mutations in these systems, and studies to define the molecular mechanisms that dictate cell-specific gene expression in relevant cell types.

NIDA: Identification of mechanisms underlying tolerance, sensitization, and addiction to drugs of abuse such as nicotine, amphetamine, cocaine, opiates, barbiturates, and hallucinogens. Identification of genetic suppressors and enhancers of the teratological effects of drugs of abuse on behavior and the nervous system. Processes involved in the development of brain regions and neurotransmitter systems mediating the hedonic and addictive properties of drugs of abuse.

NIEHS: Studies to examine the mechanism whereby environmental factors/agents alter any aspect of development. This includes the screening for mutants that ameliorate the toxicity of environmental agents, and the subsequent identification and characterization of the genes and pathways involved in their action. Characterization of the interactions among genetics, environmental agents, and time during development that lead to structural or functional abnormalities. Studies to examine the mechanistic pathways involved in developmental exposure to environmental agents and subsequent increased susceptibility to adult onset disease (developmental imprinting). Development of a mechanistically based model for testing environmental agents for developmental toxicity.

NIGMS: Development of novel methods for mutagenesis and manipulation of gene expression. Mutagenesis screens to identify and characterize genes that control fundamental biological mechanisms such as those that underlie gene regulation, chromosome organization and mechanics, cell growth and differentiation, pattern formation, sex determination, morphogenesis, cell cycle control, and behavior. Small molecule screens for phenotypes that are relevant to those fundamental biological mechanisms.

NIMH: Investigations that examine molecular, cellular, and biochemical bases of genetic mutations affecting neurogenesis, biological rhythms, learning, memory, and other cognitive functions and behaviors of the nervous system. These studies include, but are not limited to, development of screening methods for such mutations, identification, isolation, mapping, and functional analyses of the genes underlying mutations.

NINDS: Research on the development, normal function, and diseases of the nervous system. This research might include the use of mutants to understand the mechanisms controlling the following processes: neurogenesis, nervous system patterning, cell lineage, cell migration, formation of neural circuits, programmed cell death, axon pathfinding and regeneration, myelination, and motor and sensory function. In addition, the utility of mutants as models for neurodegenerative diseases for use in translational research, including therapeutic drug screens, functional neuroanatomy of the developing and adult nervous system, and use of optical imaging techniques to visualize neural activity, is of particular interest. The areas of interest listed above are not presented in any order of priority, they are only examples of areas of research to consider. Applications representing areas of interest to more than one Institute or Center will be assigned to multiple Institutes or Centers for funding consideration. Applicants are encouraged to propose work in other areas that are related to the objectives and scope of this PA.

This funding opportunity will use the NIH Individual Research Project Grant (R01) award mechanism(s). As an applicant, you will be solely responsible for planning, directing, and executing the proposed project.

This funding opportunity uses just-in-time concepts. It also uses the modular as well as the non-modular budget formats (see http://grants.nih.gov/grants/funding/modular/modular.htm). Specifically, if you are submitting an application with direct costs in each year of $250,000 or less, use the modular budget format described in the PHS 398 application instructions. Otherwise follow the instructions for nonmodular research grant applications.

The PHS 398 application instructions are available at http://grants.nih.gov/grants/funding/phs398/phs398.html in an interactive format. For further assistance contact GrantsInfo at 301-435-0714, (telecommunications for hearing impaired: TTY 301-451-0088) or by e-mail: GrantsInfo@nih.gov.

Applications must be prepared using the most current PHS 398 research grant application instructions and forms. Applications must have a D&B Data Universal Numbering System (DUNS) number as the universal identifier when applying for Federal grants or cooperative agreements. The D&B number can be obtained by calling 866-705-5711 or through the web site at http://www.dnb.com/us/. The D&B number should be entered on line 11 of the face page of the PHS 398 form.

Letters of intent are requested but not required; the letter of intent deadline for the latest cycle has passed. Letters of intent for future cycles are due August 19, 2006, 2007. Applications are due September 19, 2005, 2006, 2007. The complete version is available at http://grants.nih.gov/grants/guide/pa-files/PAR-05-080.html.

Contact: Lorette Javois, Center for Developmental Biology and Perinatal Medicine, National Institute of Child Health and Human Development, 6100 Executive Boulevard, Room 4B01, MSC 7510, Bethesda, MD 20892-7510 USA, Rockville, MD 20852 USA (for express/courier service; non-USPS service) 301-496-5541, fax: 301-480-0303, e-mail: lj89j@nih.gov.

Reference: PAR No. PAR-05-080

## Innate Immunity to NIAID Category B Protozoa

This initiative will support basic research to define the mechanisms of action by which the innate immune system recognizes and responds to the food and water-borne eukaryotes classified as NIAID Category B priority protozoan pathogens (*Cryptosporidium parvum*, *Cyclospora cayetanensis*, *Giardia lamblia*, *Entamoeba histolytica*, *Toxoplasma*, and *Microsporidia*) (http://www2.niaid.nih.gov/Biodefense/bandc_priority.htm).

The NIH and other agencies in the Department of Health and Human Services (DHHS) are currently supporting extramural research to develop new products to protect the public from the health consequences of biological agents that might be used in acts of terrorism or war. The research supported by this RFA will contribute to meeting the goals for host defense described in the NIAID Strategic Plan for Biodefense, which is located at: http://www2.niaid.nih.gov/Biodefense/Research/strat_plan.htm, by increasing our understanding of the mechanisms by which the innate immune system recognizes, responds to, and neutralizes the complex defense systems of protozoan pathogens. The complexity of the interactions between the host innate immune system and protozoan pathogens requires in-depth knowledge of innate immunity, mucosal immunity, and protozoan microbiology/biochemistry.

There is evidence that the innate immune system recognizes and responds to protozoan parasites. Recent studies have shown that *Toxoplasma gondii* stimulates host cells through TLR2 and TLR4, activates B lymphocytes, NK and NKT cells, and stimulates Interferon-gamma and NO production. In other studies, *Cryptosporidium parvum* infection activated both CD4+ and CD8+ gamma/delta T cells, and *Giardia* species stimulated the release of several effector molecules, including defensins, cryptdins, and indolicidin. Utilizing the NIAID Category B protozoa to better understand the innate immune response to eukaryotic pathogens may lead to new broad spectrum immunotherapeutics and adjuvants for protozoan vaccines.

Eukaryotic pathogens are a serious public health problem in developing countries. Exposure to pathogens such as *Giardia*, *Cryptosporidium*, and *Entamoeba* has been greatly diminished in the U.S. due to effective regulation of public water supplies and commercial food processing. Thus, interest in protozoan vaccine development has lagged behind that for prokaryotic pathogens. With the recently increased threat of biowarfare, the potential for adulteration of U.S. food and water supplies has increased. In-depth understanding of innate immune responses to protozoa is important because their genomic complexity affords them a greater variety of immune evasion mechanisms than those displayed by prokaryotes.

The major goal of this RFA is to support research focused on the mechanisms of action by which the mammalian innate immune system responds to the food and waterborne protozoa from the NIAID Category B Priority Pathogens list. Studies utilizing human cells or clinical samples are not required, but are strongly encouraged. Appropriate areas of research include, but are not limited to: 1) Characterization of host cells involved in the innate immune response to protozoa; 2) Identification of novel pathogen-associated molecular pattern recognition receptors on host cells; 3) Characterization of mediators of innate immunity that are produced by host cells stimulated by protozoa; 4) Elucidation of the intracellular signaling pathways in the mammalian innate immune cells that are stimulated by protozoa; 5) Comparison of human versus animal model molecular responses to protozoan pathogens or their components; 6) Human or animal model gene mutations or polymorphisms associated with distinctive innate immune responses to protozoa.

This initiative will unite interdisciplinary teams of researchers with expertise in innate immunity, mucosal immunity, and protozoan microbiology to stimulate scientifically sound, original research that will advance the field and encourage productive interactions among Principal Investigators. In the long term, these efforts will form a basis for the rational design of pathogen- or class-specific immunotherapeutics, as well as adjuvants that will enhance the future development of vaccines against potential bioweapons.

In some cases, immunological mechanisms relevant to biodefense are broadly applicable for many pathogens and may be most efficiently studied using model systems. Immunological research that is directed at protozoa other than those listed as NIAID Category B Priority Pathogens is responsive to this announcement if the research specifically addresses a practical approach to inducing, controlling, or improving the effectiveness of the innate immune response to NIAID Category B protozoan infection. Applicants must justify how the proposed research is applicable to immune responses against the listed agents.

Note: This RFA will NOT support clinical trials; applications requesting support for clinical trials will be considered nonresponsive and will be returned to the applicant without review. However, utilization of human-derived material in studies is considered responsive.

This funding opportunity will use the individual Research Project Grant (R01) and Exploratory/Developmental Research Grant (R21 ) award mechanisms.

This funding opportunity uses just-in-time concepts. It also uses the modular as well as the nonmodular budget formats (see http://grants.nih.gov/grants/funding/modular/modular.htm). Specifically, if you are submitting an application with direct costs in each year of $250,000 or less, use the modular budget format described in the PHS 398 application instructions, available at http://grants.nih.gov/grants/funding/phs398/phs398/398.html in an interactive format. Otherwise, follow the instructions for nonmodular research grant applications. For further assistance contact GrantsInfo, 301-435-0714, (telecommunications for the hearing impaired: TTY 301-451-0088) or by e-mail: GrantsInfo@nih.gov.

Applications must be prepared using the most current PHS 398 research grant application instructions and forms. Applications must have a D&B Data Universal Numbering System (DUNS) number as the universal identifier when applying for Federal grants or cooperative agreements. The D&B number can be obtained by calling 866-705-5711 or through the web site at http://www.dnb.com/us/. The D&B number should be entered on line 11 of the face page of the PHS 398 form.

The deadline for receipt of letters of intent is October 24, 2005, with November 22, 2005 the deadline for receipt of applications. The complete version of this RFA is available at http://www.niaid.nih.gov/ncn/budget/QA/fra-05-042.htm.

Contact: David B. Winter, Division of Allergy, Immunology, and Transplantation, National Institute of Allergy and Infectious Diseases, Room 3014, MSC-6601, 6610 Rockledge Drive, Bethesda, MD 20892-6601 USA, (for express mail use 20817), 301-496-7551, fax: 301-480-2381, e-mail: dwinter@niaid.nih.gov. Reference RFA-AI-05-042

